# Factors influencing occurrence of peritonitis in Saudi children on peritoneal dialysis

**DOI:** 10.1186/s12887-020-1936-2

**Published:** 2020-01-29

**Authors:** Khamisa Al Mokali, Zahra Al Sannaa, Faten Al Mutairi, Anwar E. Ahmed

**Affiliations:** 1 Division of Nephrology, Department of Paediatrics, King Abdullah Specialist Children’s Hospital, Ministry of the National Guard - Health Affairs City, Riyadh, Saudi Arabia; 2 King Abdullah International Medical Research Center, Riyadh, Saudi Arabia; 3 Qatif Central Hospital, Al Qatif, Saudi Arabia; 4 Maternity and Children Hospital, Madina Al Munawara, Saudi Arabia; 5 F. Edward Hébert School of Medicine, Department of Preventive Medicine & Biostatistics, Uniformed Services University of the Health Sciences, 4301 Jones Bridge Rd, Bethesda, MD 20814 USA; 6 Henry M Jackson Foundation for the Advancement of Military Medicine, Bethesda, MD USA

**Keywords:** Peritonitis, Peritoneal dialysis, Acute kidney injury

## Abstract

**Background:**

The peritonitis rate among children treated with peritoneal dialysis (PD) has not been widely reported in Saudi Arabia. The study aim was to estimate the peritonitis rate per patient-year and investigate the factors associated with higher peritonitis rates in a sample of PD children at King Abdullah Specialist Children’s Hospital-Riyadh (KASCH-R), Saudi Arabia.

**Methods:**

This retrospective cohort study included 27 PD children treated between September 2007 and December 2017 at KASCH-R. We recorded the children’s demographic and clinical data, and the frequency of peritonitis.

**Results:**

The 27 PD children reviewed (63% girls; mean age = 7.32 years old; range, 1–14 years), resulted in 86 peritonitis diagnoses in which the overall recurrence rate (in at least one episode) was 58/86 (67.4%) with a 95% confidence interval (CI), 56.5 to 77.2%. The rate of peritonitis episodes per patient-year was 0.76 (1 episode per 1.31 patient-year). The generalized Poisson model identified older children (age >  10 years) (adjusted rate ratios [aRR] = 7.273, 95% CI: 1.562–33.860), congenital nephrosis (aRR = 4.677, 95% CI: 1.443–15.155), height below 3rd percentile (aRR = 4.689, 95% CI: 1.874–11.735), weight below 3rd percentile (aRR = 5.388, 95% CI: 1.678–17.302), low albumin level (aRR = 4.041, 95% CI: 2.053–7.956), two-week duration of antibiotic therapy (aRR = 2.947, 95% CI: 1.163–7.468), which were independently associated with a high peritonitis rate.

**Conclusions:**

This study showed a high peritonitis rate in our center. Older children, congenital nephrosis, height and weight below the 3rd percentile, low albumin level, and long duration of antibiotic therapy were associated with a higher rate of peritonitis. An optimal peritonitis prevention strategy or best-practice guideline is needed to reduce and prevent peritonitis occurrence in our center.

## Background

Peritonitis is a frequent infectious complication among children on peritoneal dialysis (PD) therapy [1]. It remains as one of the main causes of patients’ early technique failure [1], hospitalization [2], morbidity [3], and mortality [3] among children on PD therapy. The peritonitis rate among children on PD therapy has been widely reported: 0.069 in Greece [4], 0.35 in South Africa [5], 0.43 in Brazil [1], 0.43 in Korea [6], 0.71 in Australasia [7], 0.75 in Tunisia [8], and 0.82 in Austria [9] in terms of episodes/patient-year. Previous studies indicate that the peritonitis incidence rate may depend on the reporting country or population [4, 9].

Although data on the peritonitis rate among PD children in Saudi Arabia are limited, the incidence of peritonitis among children on PD therapy is relatively high. The rate of peritonitis was 0.59 (1 episode/20.3 treatment months) in Taif [10] and 0.75 (1 episode/9 treatment months) in Riyadh [11] episodes/patient-year. According to these studies, factors associated with a high risk of peritonitis among children remain unrecognized in the Saudi population. Identifying the factors of peritonitis in our center may help reduce the frequency of peritonitis by targeting children at higher risk of peritonitis.

In this study, we tested the hypothesis that certain demographic and clinical factors may be associated with the high frequency of peritonitis in children end-stage renal disease (ESRD) patients treated with PD children in our center. This study aimed to estimate the peritonitis rate per patient-year and investigate the factors associated with the higher peritonitis rate in a sample of PD children at King Abdullah Specialist Children’s Hospital-Riyadh (KASCH-R), Saudi Arabia.

## Methods

This is a retrospective cohort study of ESRD children patients who are on continuous cycling PD and who were diagnosed with peritonitis from September 1, 2007 to December 31, 2017. The study was conducted in the department of Pediatrics, Division of Nephrology at KASCH-R, Saudi Arabia. KASCH-R is part of the King Abdul Aziz Medical City-Riyadh (KAMC-R), Ministry of National Guard-Health Affairs (MNGHA). The medical city also has a large university, King Saud bin Abdulaziz University for Health Sciences (KSAU*-*HS), and a research center, King Abdullah International Medical Research Center (KAIMRC). It is a government entity that serves all employees of the MNGHA and their dependents.

The study included ESRD children on PD with the diagnosis of peritonitis during the study period whose age was between 1 and 14 year old. The exclusion criteria were as follows: *(a)* age below 1 year or older than 14 year and *(b)* children with ESRD but not on PD. The study’s ethical approval was obtained from the local Institutional Review Board (IRB) at the MNGHA, with approval number RC 18/037. The consent requirement has been waived for this study due to the retrospective review.

Data were gathered, entered, and reviewed for quality by two medical residents. The diagnosis of peritonitis was based on the International Society for Peritoneal Dialysis (ISPD) [12] as defined by the presence of at least two of the following: (1) clinical features consistent with peritonitis, i.e. abdominal pain and/or cloudy dialysis effluent; (2) dialysis effluent white cell count > 100/μL or > 0.1 × 109/L (after a dwell time of at least 2 h), with > 50% polymorphonuclear; and (3) positive dialysis effluent cultures. According to ISPD, the peritonitis episodes have been descripted as: 1) recurrent peritonitis is an episode that occurs within 4 weeks of completion of therapy of a prior episode but with a different organism, 2) relapsing peritonitis is an episode that occurs within 4 weeks of completion of therapy of a prior episode with the same organism or one sterile episode, and 3) repeated peritonitis is an episode that occurs more than 4 weeks after completion of therapy of a prior episode with the same organism.

Data were retrieved from the BestCare system [13, 14] and medical records. A number of potential factors for peritonitis were gathered for analysis: patient’s age, gender, residency (Riyadh or outside Riyadh), socioeconomic status (low vs. average/high), diagnosis, glucose concentration, catheter removal, height, weight, urine output, white blood cells, neutrophils, peritoneal fluid cell counts, peritoneal analysis neutrophils, peritoneal fluid cultures, albumin level, antibiotic type, and duration of antibiotic. A total 27 PD children were identified and the number of recurrences was recorded for each PD child during the study period. The study outcome was the number of peritonitis recurrences (0, 1, 2, etc), where 0 refers to no recurrence, 1 refers to one recurrence, 2 refers to two recurrences and so on.

### Statistical analysis

**Fig. 1:**
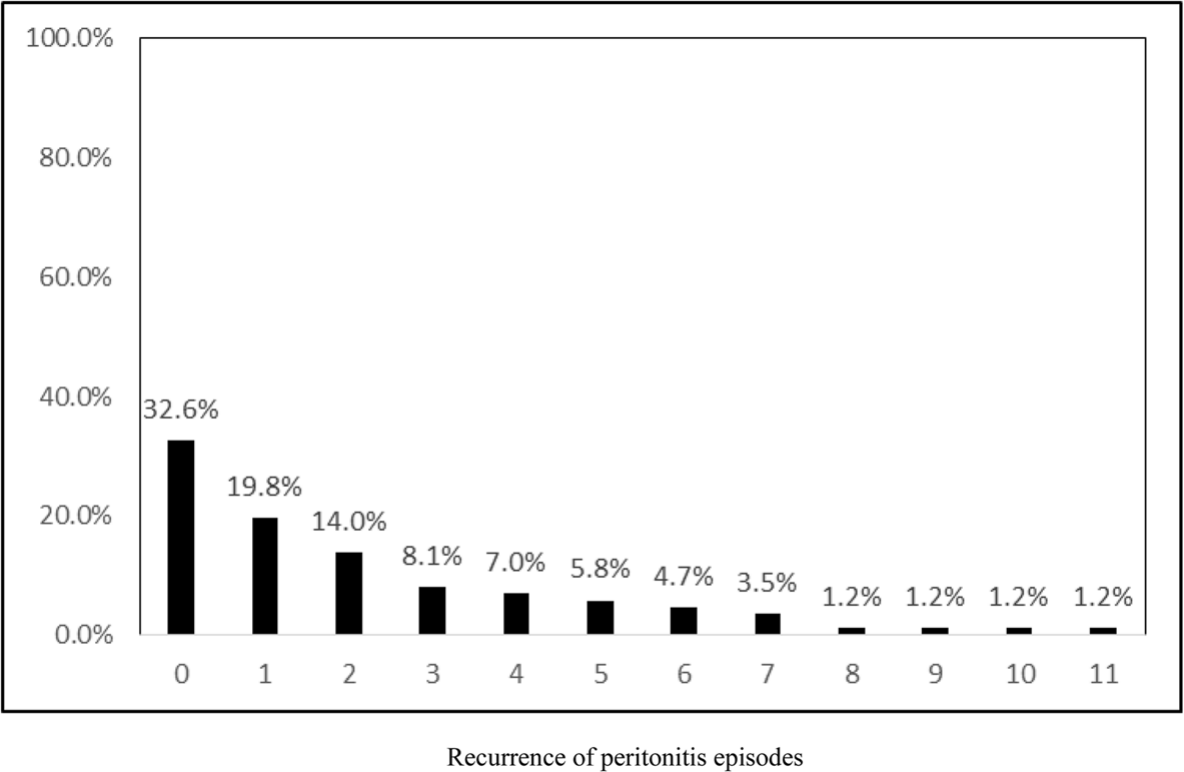
Percentage (%) of peritonitis recurrence among children on peritoneal dialysis, 0 indicates no recurrence. Recurrence of peritonitis episodes

Data analysis was conducted using SAS*®* version 9.4 (SAS Institute, Inc., Cary, North Carolina). The descriptive statistics were used to describe characteristics of diagnoses of peritonitis among children undergoing peritoneal dialysis and are presented in Table 1. Recurrent peritonitis rates among children undergoing peritoneal dialysis are illustrated by Bar chart (Fig. 1). The outcome of this study was that the number of peritonitis occurred during a 10-year period on a cohort of children undergoing peritoneal dialysis. Since we noted multiple peritonitis diagnoses on the same children, we used a generalized Poisson model to estimate the peritonitis rate while adjusting for the nonindependence. Unadjusted (Table 2) and adjusted (Table 3) rate ratios (RR and aRR, respectively) were calculated by fitting a generalized Poisson model for frequency of peritonitis as a discrete or count variable. The rate ratios and 95% confidence intervals (CI) were used to assess the strength of the associations. The Wald Chi-Square test was used to compare the frequency of peritonitis between categories of each factor. Factors with a *p*-value (P) of less than 5% were considered significantly associated with a higher peritonitis rate.

**Table 1:** Characteristics of 27 children on peritoneal dialysis

Characteristics	*Category*	n	%
Gender	*Male*	10	37.0
	*Female*	17	63.0
Age	*< 6 yrs*	11	40.7
	*6–10 yrs*	10	37.0
	*> 10 yrs*	6	22.3
Residency	*Riyadh*	15	55.6
	*Outside Riyadh*	12	44.4
Socioeconomic status	*Average/High*	24	88.9
	*Low*	3	11.1
Diagnosis	*Congenital nephrosis*	12	44.4
	*Reflux nephropathy with neurogenic bladder*	4	14.8
	*Hypoplastic kidney*	3	11.2
	*Others*	8	29.6

**Table 2:** Unadjusted factors contributing to high peritonitis rate among children on peritoneal dialysis

						95% CI for RR	
Factors	Reference	B	SE	P	RR	Lower	Upper
Female	Male	1.977	0.595	0.001*	7.218	2.247	23.184
Age: 6–10 yrs	< 6 yrs	−0.667	1.092	0.542	0.513	0.060	4.364
Age: > 10 yrs	< 6 yrs	− 0.071	1.219	0.953	0.931	0.085	10.151
Residency: Outside Riyadh	Riyadh	1.118	0.899	0.214	3.060	0.525	17.823
Low socioeconomic	Average or high	2.308	1.114	0.038*	10.051	1.134	89.128
Diagnosis							
Congenital nephrosis	Others	1.896	0.830	0.022*	6.658	1.308	33.886
Hypoplastic kidney	Others	1.462	0.893	0.102	4.313	0.750	24.808
Reflux nephropathy with neurogenic bladder	Others	−0.182	0.434	0.675	0.834	0.357	1.950
Glucose concentration							
2.50%	1.50%	1.417	1.074	0.187	4.123	0.503	33.807
Mixed (1.5–2.5%)	1.50%	0.550	0.419	0.189	1.733	0.763	3.939
Catheter removal	No	0.749	0.682	0.272	2.115	0.555	8.055
Height: below 3rd percentile	Appropriate	1.680	0.658	0.011*	5.366	1.478	19.476
Weight: below 3rd percentile	*3rd*-95th percentile	1.858	0.661	0.005*	6.408	1.754	23.413
Urine output: Anuria	Passing	1.850	0.638	0.004*	6.360	1.822	22.199
Abnormal WBC	Normal (4–12 g/l)	0.488	0.409	0.232	1.630	0.731	3.633
Neutrophils: High	Normal	1.761	0.678	0.009*	5.816	1.541	21.941
Peritoneal cell count: 100 or less	More than 100	1.517	2.293	0.508	4.557	0.051	407.810
Peritoneal analysis: More than 50	50 or less	1.970	0.556	0.001*	7.173	2.413	21.323
Peritoneal fluid culture							
Coagulase-negative staph	No growth	1.109	0.441	0.012*	3.030	1.277	7.189
Viridans streptococci	No growth	0.913	0.849	0.282	2.491	0.472	13.147
Others	No growth	2.217	0.787	0.005*	9.179	1.964	42.899
Albumin level: Low	Normal	1.647	0.668	0.014*	5.189	1.402	19.215
Antibiotic type							
Cefazolin	Mixed(cefazolin+ceftazidime)	−0.084	0.726	0.908	0.919	0.222	3.815
Ceftazidime	Mixed(cefazolin+ceftazidime)	0.906	0.733	0.217	2.475	0.588	10.419
Vancomycin	Mixed(cefazolin+ceftazidime)	1.005	0.726	0.166	2.732	0.658	11.334
Other	Mixed(cefazolin+ceftazidime)	3.031	1.464	0.038*	20.723	1.177	364.880
Duration of antibiotic							
2 weeks	10 days	1.302	0.553	0.019*	3.677	1.244	10.868
3 weeks/3 months	10 days	1.500	1.224	0.220	4.482	0.407	49.349

*The Wald Chi-Square test is significant at *P* ≤ 0.05. *RR* unadjusted rate ratio. B, the estimated Poisson regression coefficient

**Table 3:** Adjusted factors contributing to high peritonitis rate among children on peritoneal dialysis

						95% CI for aRR	
Factor	Reference	B	SE	P	aRR	Lower	Upper
Intercept		−6.321	1.804	0.001			
Female	Male	0.712	0.560	0.204	2.039	0.680	6.114
Age: 6–10 yrs	< 6 yrs	−0.684	0.640	0.286	0.505	0.144	1.771
Age: > 10 yrs	< 6 yrs	1.984	0.785	0.012*	7.273	1.562	33.860
Residency: Outside Riyadh	Riyadh	0.976	0.366	0.008*	2.654	1.295	5.435
Diagnosis							
Low socioeconomic	Average or high	0.184	0.819	0.822	1.202	0.242	5.978
Congenital nephrosis	Others	1.543	0.600	0.010*	4.677	1.443	15.155
Hypoplastic kidney	Others	0.745	0.488	0.127	2.107	0.809	5.489
Reflux nephropathy with neurogenic bladder	Others	−0.035	1.130	0.976	0.966	0.106	8.842
Glucose concentration							
2.50%	1.50%	0.115	0.559	0.838	1.122	0.375	3.356
Mixed (1.5–2.5%)	1.50%	0.093	0.431	0.829	1.098	0.472	2.555
Catheter removal	No	−0.485	0.515	0.346	0.616	0.225	1.687
Height: below 3rd percentile	Appropriate	1.545	0.468	0.001*	4.689	1.874	11.735
Weight: below 3rd percentile	3rd-95th percentile	1.684	0.595	0.005*	5.388	1.678	17.302
Urine output: Anuria	Passing	−0.139	0.905	0.878	0.870	0.148	5.128
Abnormal WBC	Normal (4–12 g/l)	0.061	0.393	0.876	1.063	0.492	2.297
Neutrophils: High	Normal	0.820	0.747	0.272	2.270	0.525	9.812
Peritoneal cell count: 100 or less	More than 100	1.705	1.326	0.199	5.499	0.409	73.999
Peritoneal analysis: More than 50	50 or less	1.790	1.360	0.188	5.990	0.417	86.158
Peritoneal fluid culture							
Coagulase-negative staph (staph Epidermidids)	No growth	0.803	1.073	0.454	2.232	0.273	18.265
Viridans streptococci	No growth	1.138	0.876	0.194	3.121	0.561	17.366
Others	No growth	1.362	0.530	0.010*	3.905	1.383	11.022
Albumin level: Low	Normal	1.397	0.346	0.001*	4.041	2.053	7.956
Cefazolin	Mixed(cefazolin+ceftazidime)	−0.962	0.996	0.334	0.382	0.054	2.693
Ceftazidime	Mixed(cefazolin+ceftazidime)	−1.209	0.778	0.120	0.298	0.065	1.370
Vancomycin	Mixed(cefazolin+ceftazidime)	−0.100	0.721	0.889	0.905	0.220	3.718
Other	Mixed(cefazolin+ceftazidime)	0.735	1.051	0.484	2.085	0.266	16.341
Duration of antibiotic							
2 weeks	10 days	1.081	0.475	0.023*	2.947	1.163	7.468
3 weeks/3 months	10 days	1.885	0.689	0.006*	6.587	1.708	25.408

*The Wald Chi-Square test is significant at *P* ≤ 0.05. *aRR* adjusted rate ratio. B, the estimated Poisson regression coefficient

## Results

A total of 86 peritonitis diagnoses was reported during the period 2007–2017 from 27 ESRD children treated by PD (mean age, 7.3 ± 3.7, range: 1–14 year). There were 63% girls and 37% boys (Table 1). Over 10 years, 6 patients had permanent PD catheter removal and shifted to hemodialysis (HD). None our patients had ostomy. Concomitant exit-site infection was found in 11 episodes out of 86 of peritonitis. Overall peritonitis recurrence rate in (in at least one episode) the children studied was 58/86 (67.4%): 19.8% had 1 recurrent, 14% had 2 recurrent, 8.1% had 3 recurrent, 7% had 4 recurrent, and 18.5% had 5 or more recurrent (Fig. 1). The rate of peritonitis in our center was 0.76 episodes per patient-year (1 episode per 1.31 patient-year), 86 episodes during 112.667 years (1352 months). Of the sample, congenital nephrosis (55.8%), and low albumin level (96.5%) were common characteristics among children undergoing peritoneal dialysis in our center. There was 1 death in our sample.

Table 2 illustrates individual factors associated with the increased peritonitis rate. Female gender (unadjusted rate ratios [RR] = 7.218, 95% CI: 2.247–23.184), low socioeconomic status (RR = 10.051, 95%CI: 1.134–89.128), congenital nephrosis (RR = 6.658, 95% CI: 1.308–33.886), height below 3rd percentile (RR = 5.366, 95% CI: 1.478–19.476), weight below 3rd percentile (RR = 6.408, 95% CI: 1.754–23.413), anuric patients (RR = 6.360, 95% CI: 1.822–22.199), *coagulase-negative staph* (RR = 3.030, 95% CI: 1.277–7.189), and low albumin level (RR = 5.189, 95% CI: 1.402–19.215), were significantly associated with a high peritonitis rate.

Table 3 illustrates independent factors associated with the increased peritonitis rate. Older children (age >  10 years) (adjusted rate ratios [aRR] = 7.273, 95% CI: 1.562–33.860), outside Riyadh residency (aRR = 2.654, 95% CI: 1.295–5.435), congenital nephrosis (aRR = 4.677, 95% CI: 1.443–15.155), height below 3rd percentile (aRR = 4.689, 95% CI: 1.874–11.735), weight below 3rd percentile (aRR = 5.388, 95% CI: 1.678–17.302), other peritoneal fluid culture (aRR = 3.905, 95% CI: 1.383–11.022), duration of antibiotics 2 weeks (aRR = 2.947, 95% CI: 1.163–7.468), and 3 weeks/3 months (aRR = 6.587, 95% CI: 1.708–25.408) and low albumin level (aRR = 4.041, 95% CI: 2.053–7.956) were independently associated with a high peritonitis rate.

## Discussion

Data regarding the frequency of peritonitis and its factors among ESRD children treated by PD in the Saudi population are limited. The authors studied a sample of children with ESRD who are on continuous cycling peritoneal dialysis. The peritonitis rate was relatively high in our center as compared to ISPD recommendations [12], 0.76 episodes per patient-year, with 86 peritonitis episodes during 112.667 years (1352 months) occurring in 27 patients. Our findings confirm a previous study (0.75 episodes per patient-year) in Riyadh by Mirza et al*.* [11], where no variation was noted in the peritonitis rates among children. According to our study and their study, the peritonitis rate among Saudi children on PD appears higher than that reported in other countries [1, 4–6] where they met ISPD recommendations. There is a need for an urgent intervention program to reduce and prevent peritonitis in our center.

The study identified a number of factors associated with the high frequency of peritonitis among PD children in our center. ESRD children on PD who reside outside of Riyadh tend to have a higher peritonitis rate. In this study, 21(24.4%) of peritonitis episodes recorded for children with low socioeconomic status in which 18 (85.7%) recorded for children residing outside Riyadh city. Lack of access to specialized care providers may explain the high risk of peritonitis in patients residing outside Riyadh city. This subgroup may be further evaluated and monitored to reduce peritonitis.

A number of previous studies [15–18] reported that young ESRD children treated by PD are associated with a higher incidence of peritonitis. Unlike these studies, the current study shows that PD children who are older than 10 years of age were associated with an adjusted rate ratio of 7.273 compared to children younger than 6 years. It is possible that the older children (> 10 years) have more exposure to PD duration and thus increase the risk of peritonitis [19]. Older ESRD children (> 10 years) should be monitored to prevent peritonitis and related complications.

In this study, longer antibiotic therapy was significantly associated with a higher incidence of peritonitis. This association is probably affected by antibiotic regimens, and further study is needed to assess the impact of specific antibiotic regimens on the incidence of peritonitis. Low serum albumin was significantly associated with an increased incidence of peritonitis in our population. Serum albumin is a marker for malnutrition and is one of the factors predisposing to infection in uremic and dialysis patients [20]. Weight and height at the time of dialysis were associated with a higher rate of peritonitis. Weight and height below the 3rd percentile were found to be 72.1 and 88.2%, respectively.

In this study, the authors found that *viridans streptococci* is the commonest causative organism of peritonitis in PD patients, as this observation had been reported previously in different studies [18, 21]. However, *coagulase-negative staph* was the most common gram positive organism which could be related to hand hygiene. Mupirocin application at the exit site significantly lowers the incidence of staph aureus exit site infections and peritonitis due to staph aureus [22, 23]. The routine use of mupirocin on a daily basis for all of our patients in our center may explain the low rate of staph aureus peritonitis in only 2 episodes (2.3%). Gram negative peritonitis may require further studies.

The study does have limitations. Significant findings must be investigated carefully as it indicates association. This is a single-center study, and generalization may be limited to the study population.

The authors noticed a number of limitations. The peritonitis data was collected from a single center, thus the study may not include peritonitis episodes reported to other health facilities. The recruitment over a 10 year period may bias the results as guidelines changed during this time. All patients had been started on PD within 14 days of catheter insertion so, the authors were not able to assess the risk of early (< 14 days post placement) versus delayed use (> 14 days) of catheter. Twenty-seven patients with peritonitis or the entire cohort of patients receiving peritoneal dialysis. The calculation of peritonitis rate was performed using the total duration of antibiotic rather than the entire cohort of patients receiving peritoneal dialysis. The authors were not able to collect data on factors that could impact peritonitis recurrences in children undergoing PD such as catheter implantation (by nephrologist or surgeon), caregiver or patient training time, use of antimicrobial implant prophylaxis, predialysis care, and type of PD. No control group was used to compare the rate of peritonitis and small sample size can be additional limitations to the study.

Despite these limitations, this is the first study identifying independent factors for peritonitis in ESRD children on PD in the Saudi population. A large multi-center study is needed to establish a national peritonitis rate among ESRD children treated by PD in Saudi Arabia. Establishing peritonitis registries with continuous quality assessment in Saudi Arabia for ESRD children on PD may be a priority to reduce and prevent peritonitis and other unfavorable outcomes.

## Conclusion

This study showed a high peritonitis rate in our center. Older children, congenital nephrosis, height and weight below the 3rd percentile, low albumin level, and long duration of antibiotic therapy were associated with a higher rate of peritonitis. An optimal peritonitis prevention strategy or best-practice guideline is needed to reduce and prevent peritonitis occurrence in our center.

## Data Availability

The health records for this study can be retrieved from the Ministry of National Guard - Health Affairs.

## Abbreviations

aRR: Adjusted rate ratios
CI: Confidence interval
ESRD: End-stage renal disease
HD: Hemodialysis
IRB: Institutional Review Board
ISPD: International Society for Peritoneal Dialysis
KAMC-R: King Abdul Aziz Medical City-Riyadh
KASCH-R: King Abdullah Specialist Children’s Hospital-Riyadh
KSAU-HS: King Saud bin Abdulaziz University for Health Sciences
MNGHA: Ministry of National Guard-Health Affairs
PD: Peritoneal dialysis
